# Editorial: Probiotics - A New Tool for Restraining Infectious Pathogens and Antibiotic Resistance

**DOI:** 10.3389/fcimb.2022.938282

**Published:** 2022-06-29

**Authors:** Fanglei Zuo, Guorong Liu, Junhua Jin

**Affiliations:** ^1^ Department of Biosciences and Nutrition, Karolinska Institutet, Huddinge, Sweden; ^2^ Beijing Engineering and Technology Research Center of Food Additives, Beijing Technology and Business University, Beijing, China; ^3^ Food Science and Engineering College, Beijing University of Agriculture, Beijing Laboratory of Food Quality and Safety, Beijing Key Laboratory of Detection and Control of Spoilage Organisms and Pesticide Residues in Agricultural Products, Beijing, China

**Keywords:** probiotics, pathogens, antimicrobials, antibiotic resistance, live biotherapeutics

Antibiotic resistance is one of the biggest threats to global health, food security, and development today. Indiscriminate use of antibiotics in humans and animals is accelerating the process. Alternative therapy by probiotics to address certain health conditions in both animals and humans may alleviate antibiotic selective pressures on microorganisms and contribute to reducing the emergence of antibiotic-resistant pathogens. Little is known about the mechanisms of action of probiotics, however, the recent expansion of genome editing tools such as CRISPR-Cas technologies available for probiotic bacteria will significantly advance our understanding of the molecular mechanisms by which probiotics antagonize pathogens, thereby contribute to the development of novel prophylactic and therapeutic agents against infections. These technologies together with other synthetic biology approaches, Omics technologies, and computational bioinformatics will greatly advance our understanding of the role of probiotics in the maintenance of human health and boost the development of next-generation live biotherapeutics, which may contribute to reducing the global burden of antibiotic resistance.

The present Research Topic has gathered four articles that summarize the role of bacterial metabolite short-chain fatty acids (SCFAs) in the protection against infections and probiotics in managing drug-resistant fungal infections, investigate the anti-biofilm and immunomodulatory effects of cell free filtrate (CFF) of *Lactobacillus acidophilus* ATCC 4356, and the ability of *Bacillus subtilis* LF11 to protect the intestinal epithelium against *Salmonella* infection.

Schlatterer et al. have summarized recent advances in understanding the role of SCFAs and their host receptor, the seven-transmembrane G-protein-coupled receptors (GPCRs) FFAR2, in various infection types, and further proposed an additional therapeutic strategy to fight infections by manipulating FFAR2-mediated interaction of SCFAs with host innate immune cells. Many evidences demonstrated that activation of FFAR2 by SCFA administration diminishes the susceptibility toward various types of infections caused by bacteria and viruses. Although the exact molecular mechanisms and outcome of SCFA-FFAR2 modulation are currently under debate, the author suggested that SCFA administration during infectious disease or the intake of a high-fiber diet to enhance microbiome SCFA production could be a strategy to treat and prevent infections.

Fungi, like bacteria, can also develop antibiotic resistance. The prevalence of drug-resistant fungal infections is increasing and becoming an urgent health threat. Wu et al. reviewed the progress of probiotics as a new strategy for the treatment of fungal infections. Although the molecular mechanisms underlying the inhibitory effect of probiotics on fungi remains not fully understood, probiotics do show strong antifungal effects probably through inhibition of filamentation, biofilm formation, and interfering with cell attachment. The authors indicated that probiotics can potentially be developed as novel therapeutics against fungal infections, either administrated alone or in combination with traditional antifungal drugs.

Wilson et al. studied the effects of different concentrations of *L. acidophilus* ATCC 4356 secreted products on both monocyte/macrophage activation as well as *Pseudomonas aeruginosa* growth and biofilm formation/removal. The authors found that different concentrations of *L. acidophilus* ATCC 4356 CFF possess variable bactericidal, anti-biofilm, and immunomodulatory effects. The study highlighted the importance of evaluating different concentrations of *L. acidophilus* CFF to be used *in vivo* for different and specific conditions.

Zhang et al. investigated the protection role of *Bacillus subtilis* LF11 against *Salmonella* infections. The authors showed that *B. subtilis* LF11 inhibits adhesion to and invasion of intestinal epithelial cells by *S. braenderup* H9812, enhances the expression of the tight junction proteins, and attenuates the inflammatory response of NCM460 cells caused by *S. braenderup* H9812. These findings support the potential of *B. subtilis* LF11 to be developed as an antibiotic alternative to prevent enteric diseases in broilers.

## Author Contributions

All authors listed have made a substantial, direct, and intellectual contribution to the work and approved it for publication.

## Funding

This work was supported by Karolinska Institutet Research Foundations Grants (FS-2020:0007 to FZ), Ruth and Richard Julin Foundation (FS-2021:0003 and FS-2022:0003 to FZ), Tore Nilsons Stiftelse För Medicinsk Forskning (2021-00879 to FZ), magn bergvalls stiftelse (2021-04218 to FZ).

## Conflict of Interest

The authors declare that the research was conducted in the absence of any commercial or financial relationships that could be construed as a potential conflict of interest.

## Publisher’s Note

All claims expressed in this article are solely those of the authors and do not necessarily represent those of their affiliated organizations, or those of the publisher, the editors and the reviewers. Any product that may be evaluated in this article, or claim that may be made by its manufacturer, is not guaranteed or endorsed by the publisher.

